# Mode of Inserting Partial Sets

**Published:** 1856-12

**Authors:** F. A. Brewer

**Affiliations:** Fulton, Mo.


### MODE OF INSERTING PARTIAL SETS.

Messrs Editors:—As you requested me to send for publication in the Register any practical hints that might suggest themselves to my mind, I will comply with your request so far as to trouble you with a few lines concerning a plan which I adopted about a year ago, and which I presume some other dentists are also using. I find it of great use, in inserting partial sets of teeth.

My plan is this:—I take an impression, and proceed as usual, till the plate is struck up. Then, without attaching clasps, I try it in the mouth, and, if it fits properly, I let it remain, and take another impression over the plate. On taking this impression out of the mouth, the plate accompanies it, and, without removing it, I fill the imprsssion with plaster. On removing the wax, the plate is found on the plaster model, exactly as it sets in the mouth. I next fit the clasps, and without soldering, try them in the mouth, and mark their positions, in relation to the plate, with a sharp instrument. I then remove them from the mouth, and attach them on the cast, mark for mark, and then solder them as they sit on the plaster model. I spoil the first and second models, but deem the advantage gained more than a compensation for the expense and trouble, as I thus have a beautiful fit. I then fit it carefully in the mouth, the clasps being soldered, and take another impression over it, and then attach teeth and solder in the usual way. F. A. BREWER.

*Fulton, Mo., Oct.* 21*st*, 1856.

